# LIM Protein Ajuba Participates in the Repression of the ATR-Mediated DNA Damage Response

**DOI:** 10.3389/fgene.2013.00095

**Published:** 2013-05-28

**Authors:** Sampada Kalan, Anastasiya Matveyenko, Diego Loayza

**Affiliations:** ^1^Department of Biological Sciences, Hunter College, New York, NY, USA

**Keywords:** LIM protein, Ajuba, ATR, DNA damage, RPA

## Abstract

LIM proteins constitute a superfamily characterized by the presence of a LIM domain, known to be involved in protein–protein interactions. Our previous work has implicated members of the Zyxin family of LIM proteins, namely TRIP6 and LPP, in the repression of the DNA damage response (DDR) at telomeres. Here, we describe a role for Ajuba, a closely related LIM molecule, in repressing the ATR-mediated DDR. We found that depletion of Ajuba led to apparent delays in the cell cycle, accompanied with increased Rb phosphorylation, Chk1 phosphorylation, induction of p53, and cell death. Ajuba could be found in a complex with replication protein A (RPA), and its depletion led to RPA phosphorylation, known to be an early event in ATR activation. We propose that Ajuba protects against unscheduled ATR signaling by preventing inappropriate RPA phosphorylation.

## Introduction

Maintenance of genomic integrity is essential for accurate transmission of genetic information and cell viability. DNA damage by endogenous and exogenous agents can lead to genomic instability, itself a causative factor in early human tumorigenesis (Bartkova et al., 2005; Gorgoulis et al., 2005). Cells have specific checkpoints to detect damaged or abnormally structured DNA and allow for activation of repair mechanisms, or activation of apoptosis (reviewed in Ciccia and Elledge, 2010). Checkpoints operate at distinct points in the cell cycle to check for DNA lesions and act to delay transitions from both G1 to S phase and G2 to M phase, as well as within S (reviewed in Zhou and Bartek, 2004). In addition, checkpoints can monitor cells for M phase exit. In order to counteract and repair the DNA damage, the cell elicits a DNA damage response (DDR), under the control of signaling kinases part of the PIKK family, ATM, ATR, and DNA-PK (Ciccia and Elledge, 2010). These DDRs are organized pathways consisting of specific steps of damage sensing, transduction of a damage signal, and induction and recruitment of repair proteins to the damaged sites. Of those, ATM is important for the repair of double strand breaks and is not essential for viability, but controls an important tumor suppressor pathway. ATR however, is essential, and is activated upon types of damage generated by UV irradiation, such as Thymine dimers, or DNA replication defects in S phase such as replication fork collapses or accumulation of single stranded DNA. It has been proposed that the essential nature of the ATR pathway is caused by the necessity to repair spontaneous damage occurring during a normal S phase, which would lead to an intolerable level of damage if left unrepaired (see Hurley and Bunz, 2007 and references therein).

The ATM and ATR kinases have a number of target substrates, among which the kinases Chk1 and Chk2, which are important for the respective cellular responses to the damage. Chk2 is an ATM target and is phosphorylated after ionizing radiation. Chk1, on the other hand, is a direct target of ATR, and is phosphorylated during replication stress or UV irradiation. ATR, therefore, is the kinase involved in responding to endogenous lesions or errors occurring through the action of replication forks during a normal S phase (Sorensen et al., 2004; Vassin et al., 2009).

During DNA replication, the single stranded DNA produced is bound by replication protein A (RPA), a complex of three subunits, RPA70, RPA32, and RPA14, which binds single DNA through OB fold structural motifs in a sequence-independent manner (Wold, 1997). RPA is a central molecule in the activation of ATR. The RPA32 subunit is phosphorylated after damage, and recruits ATRIP, itself required for the activation process (Zou and Elledge, 2003). Upon a sustained DDR, the ATM pathway is activated leading to activation of p53 and further checkpoint delays in the cell cycle.

Another event that can trigger DDR is telomere deprotection. Mammalian telomeres consist of TTAGGG tandem repeats that end with a 3′ overhang (Palm and de Lange, 2008). A six-protein complex called shelterin binds to the telomeric repeats, and, as part of this complex, TRF2 was found to prevent inappropriate activation of ATM (Karlseder et al., 2004). Another shelterin protein, POT1, directly binds the telomeric single stranded overhang and protects against ATR activation (Lazzerini Denchi and de Lange, 2007; Palm et al., 2009).

Our laboratory has previously shown that members of a distinctive class of molecules called LIM proteins are implicated in telomere protection by repressing DDR at telomeres (Sheppard and Loayza, 2010; Sheppard et al., 2011). Specifically, LIM proteins TRIP6 and LPP belong to the Zyxin family (Kadrmas and Beckerle, 2004) and interact with the shelterin complex to prevent DDR activation at telomeres. The Zyxin family is characterized by the presence of three LIM domains present at the C-terminus, with each domain containing of two Zinc fingers, and a unique pre-LIM region at the N-terminus. They also posses a nuclear export signal close to the N-terminus and hence can shuttle between the nucleus and the cytoplasm (Wang and Gilmore, 2001). The LIM protein TRIP6, in particular, was shown to interact with OB-fold-containing protein POT1 through the C-terminal LIM domains (Sheppard and Loayza, 2010).

Here, we are investigating the role of Ajuba, a closely related LIM protein, part of the Zyxin family. We found that Ajuba also participates in the repression of the DDR, but in a genome-wide fashion. We describe the role of Ajuba as a repressor of the ATR pathway, and show that this molecule is in a complex with RPA and prevents unscheduled phosphorylation of RPA32. We propose a model in which Ajuba controls the transition between local activation of ATR during a normal S phase and the global ATR activation occurring after extensive DNA damage.

## Materials and Methods

### Cell lines and antibodies

The cell lines used were HTC75 cells and IMR90. The HTC75 cell line is a HT1080 derivative described in (van Steensel and de Lange, 1997). IMR90 cells were obtained from the ATCC at population doubling 21. The cells were grown in DMEM supplemented with 1% penicillin and streptomycin, 10% BCS for HTC75, and 10% FBS for IMR90 cells. The Ajuba antibody was obtained from Abcam (AB64451). The Ajuba serum was generated against a peptide conjugated to KLH and used for immunization into rabbits, as per the protocol set by the manufacturer (BioSynthesis, Lewisville, TX, USA). The peptide was: NH2-CPRGATGGPGDEPLEPAREQGSLDA-OH for Ajuba. The antibodies for Rb-pS807/811(9308), PARP (9542), p53-p-Ser20 (9287), Cyclin A2 (4656), Chk1-p-S345 (2348), Chk1-p-S296 (2349) were obtained from Cell signaling. The total Chk1 antibody was purchased from Sigma-Aldrich (C9358). The p53 antibody was acquired from Millipore (04-1083). The RPA-p-T21 antibody was purchased from Abcam (AB109394). The GAPDH antibody was obtained from Santa Cruz (sc-32233). The p53BP1 (NB100-304) and RPA2 (9A1) antibody was purchased from Novus.

### Depletion by siRNA

HTC75 cells and IMR90 cells were maintained in DMEM (Invitrogen)/1% penicillin and streptomycin/10% FBS. Ajuba specific siRNAs were synthesized by Dharmacon RNA Technologies. For Ajuba RNAi, double-stranded siRNA were designed to target the following sequences: Ajuba si#1 siRNA 5′-CCAAAUGGAUUGUGGAAGAUU-3′, Ajuba si#2 siRNA 5′-GGGAAAGAGGUCAGAUUUAUU-3′, and Ajuba si#3 siRNA 5′-GCAGCUGAGUGAUGAGGAAUU-3′. The cells were transfected using Lipofectamine (Invitrogen) according to the manufacturer’s instructions. Cells were grown to confluency of approximately 20–25% in a six-well plate 18–24 h prior to transfection. Transfections were done twice, once within a 24 h interval and another at 48 h. The cells were processed 72 h after the first transfection. As a control, siRNA designed to target GFP (Dharmacon) was used.

### Immunofluorescence

Immunostaining for p53BP1 performed on cells plated onto glass coverslips and processed for RNAi. After the transfection period, cells were washed twice with PBS, the cells were then fixed with PBS/3% paraformaldehyde for 10 min at RT. After two PBS washes, cells were permeabilized with PBS/0.5% NP40 and later blocked with PBG [PBS/0.2% fish gelatin. 0.5% bovine serum albumin (BSA)] for 30 min. Coverslips were then incubated with the rabbit anti-p53BP1 antibody (Novus NB100-304A-1), at a concentration of 1:500 in PBG overnight. Cover slips were then rinsed three times with PBG solution and incubated with secondary TRITC-conjugated goat anti-rabbit antibody (Jackson Immunoresearch) in PBG at a concentration of 1:500 for 45 min at RT. Cover slips were rinsed two times with PBG. Coverslips were then incubated with PBG and 4,6-diamidino-2-phenylindole (DAPI) at 100 ng/mL to visualize the nuclei. Coverslips were mounted on to slides with embedding media. Images were collected with an Olympus BX61 fluorescence microscope using a 60× objective connected to a Hamamatsu ORCA-ER CCD camera, controlled by the SlideBook 5.1 image capture software.

### Cell cycle analysis by FACS

The cells were collected and rinsed twice in cold PBS/2 mM EDTA, resuspended in 7 mL of PBS/2 mM EDTA/2% BSA, 3 mL of cold 100% Ethanol was added drop wise and the cells were kept at 4°C for 24 h fixation. The cells were then spun down and resuspended in 0.5 mL of PBS/2 mM EDTA. Ten microliters of heat inactivated RNase A (10 mg/mL) and 25 μl of Propidium Iodide (1 mg/mL) were added and the cells were incubated at 37°C for 30 min. The samples were then analyzed using a FACS Calibur Flow Cytometer.

### Co-immunoprecipitations

The immunoprecipitations were performed as described in (Loayza and de Lange, 2003).

## Results

### Depletion of Ajuba by siRNA leads to S-phase delay in HTC75 cells

**Figure 1:**
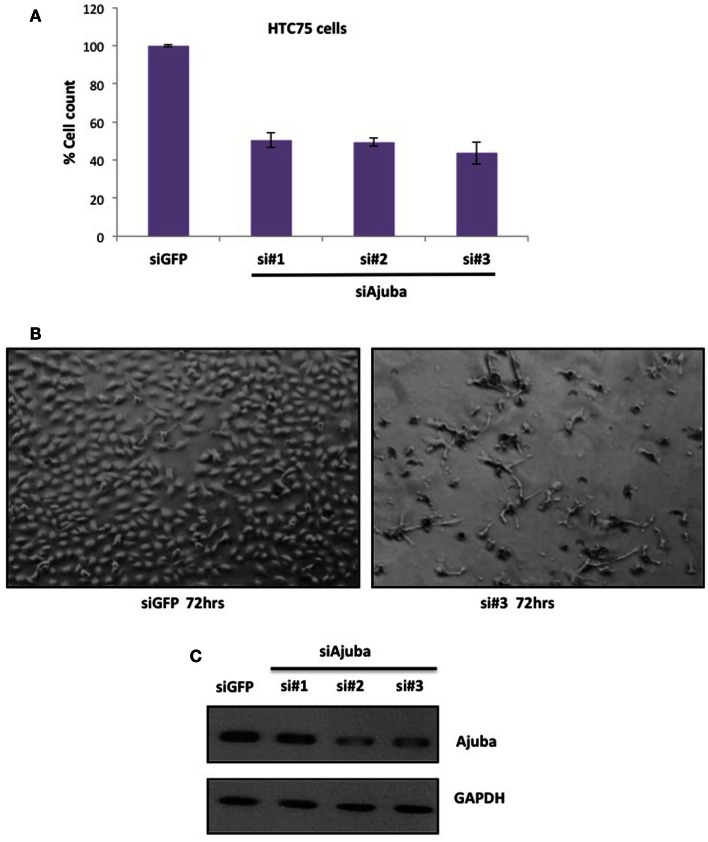
**Depletion of Ajuba in HTC75 cells results in reduced cell number**. **(A)** Quantitation of three independent siRNA experiments with cell counts performed at 72 h after the first transfection. **(B)** Cells shown 72 h after transfection, with GFP siRNA as controls. **(C)** Western blot showing the depletion of Ajuba by siRNA with three different target sites.

**Figure 2:**
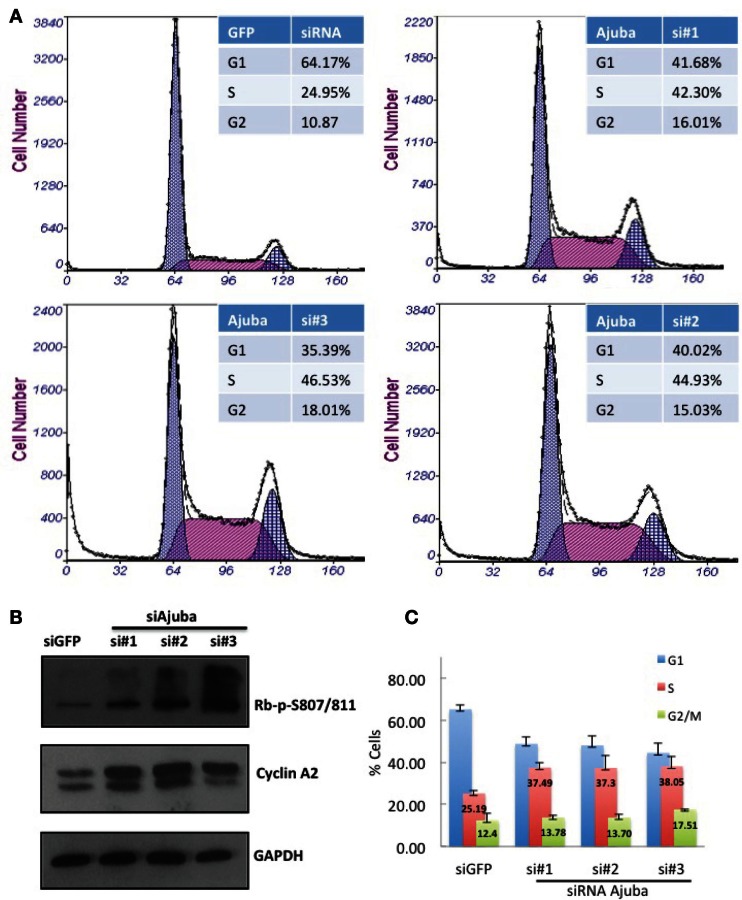
**HTC75 cells depleted for Ajuba show S-phase delay**. Attached and floating cells were processed 72 h after siRNA transfection for **(A)** FACS sorting after PI staining, and **(B)** Western blots for phosphorylated Rb or total levels of Cyclin A2, with GAPDH as a loading control. **(C)** Cell cycle profiles of cells processed as in **(A)**, with% of cells in G1 or G2/M indicated as averages of three independent experiments.

We performed siRNA depletion of Ajuba in HTC75 cells using three different target sites. The most effective depletion was observed by Western blot with siRNA #3 (Figure 1C). In all three cases, a significant reduction in total cell count was observed, down to approximately 50% of viable cells compared to the GFP siRNA control (Figures 1A,B), at 72 h after transfection. We then sought to determine whether the cells at this time point showed a specific alteration in their cell cycle profile. To this end, cells were fixed, stained with propidium iodine and processed for FACS analysis for DNA content. In all three cultures treated with Ajuba siRNA, cells exhibited a significant increase in their number in S phase: respectively 42.3, 44.9, and 46.5% in S phase in cells treated with siRNA #1,2, or 3, compared with 24.95% in the GFP siRNA control (Figure 2A). An average of three experiments solidified this observation, with siRNA #3 having the strongest effect, with 38% of cells in S phase versus 25.1% of the cells in the control siRNA (Figure 2C). We noted also a slight but reproducible increase in the number of cells in G2/M with siRNA #3: 17.5% versus 12.4% of the cells in the GFP siRNA control. There was also a notable increase in cells with sub-G1 DNA content in all three siRNA depletions compared to controls (Figure 2A). We concluded that depletion of Ajuba led to a delay in S phase, possibly due to checkpoint activation. Protein extracts were prepared from these cells in order to probe the molecular effects of Ajuba depletion. Given the cell cycle profile observed, we were particularly interested in markers characteristic of this particular phase of the cell cycle. We found that, in the viable cells, Rb was hyperphosphorylated (Figure 2B), compatible with the cells having passed the G1/S transition. Cyclin A2 was also found at high levels (Figure 2B), a feature of cells undergoing DNA replication.

### Depletion of Ajuba results in ATR activation followed by apoptosis in HTC75 cells

**Figure 3:**
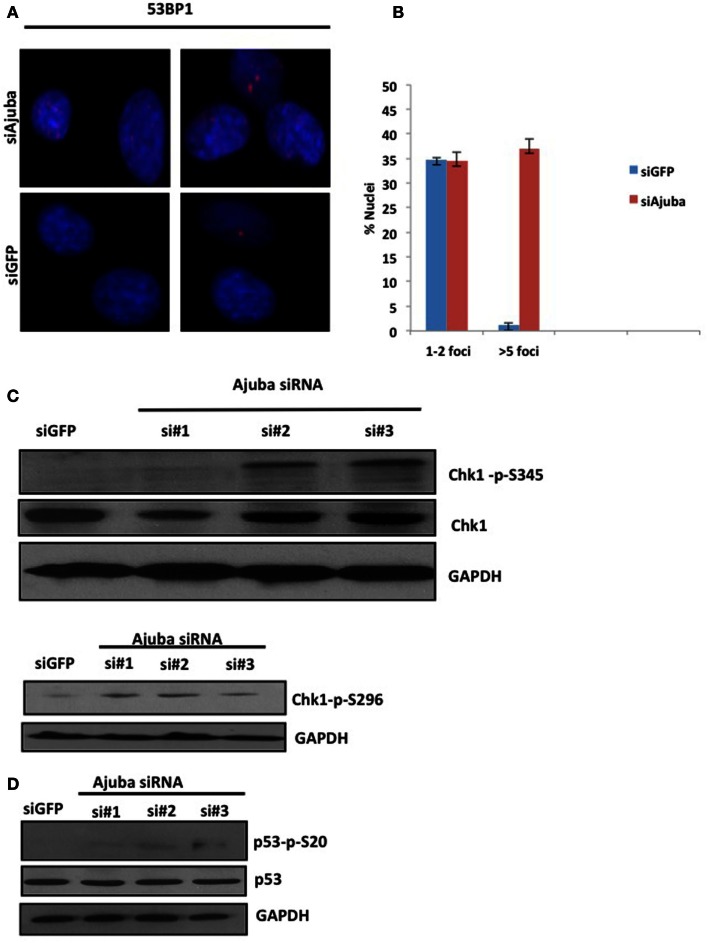
**Depletion of Ajuba results in activation of the DNA damage response**. **(A)** Staining for p53BP1 foci on cells fixed 72 h after transfection with siRNA #3, and siGFP as a negative control. Quantification of the number of p53BP1 foci on three independent siRNA experiments shown in **(B)**. (**C, D**) Western blots for induction of Chk1 phosphorylation and induction of p53 phosphorylation, showed for the three target sites, 72 h after siRNA transfection.

The delay of cells in S phase prompted us to assess the level of endogenous DNA damage in cells depleted for Ajuba. To that end, we stained for a known marker involved in DNA repair, p53BP1, which accumulates at sites of DNA damage early in the response. We found that depletion of Ajuba led to a significant increase of nuclei with more than five p53BP1 foci (Figures 3A,B), with 37% of nuclei compared to 2% of nuclei in GFP control experiments. In HTC75 cells, there is an average of two p53BP1 foci, which could be detected in control siRNAs and represented a background level in these cells. This apparent induction of the DDR in S phase likely activated a known DNA repair pathway, and in particular ATR, sensitive to DNA replication stress. Indeed, there was activation of ATR following Ajuba depletion, as observed by phosphorylation of Chk1 at residues Ser-345 and Ser-296, which are both ATR-dependent (Liu et al., 2000; Okita et al., 2012) (Figure 3C). In accordance with this finding, p53 was also weakly activated by Ajuba depletion (Figure 3D), as observed by Ser20 phosphorylation associated with siRNA #3. It is possible that this effect is due to activation of ATM as a subsequent effect, although we could not detect perceptible Chk2 phosphorylation (not shown). In all cases, the effects were again best seen with siRNA#3. Altogether, these results support the conclusion that Ajuba depletion led to phosphorylation of Chk1 and activation of p53, suggesting a role for Ajuba in repressing the ATR pathway.

**Figure 4:**
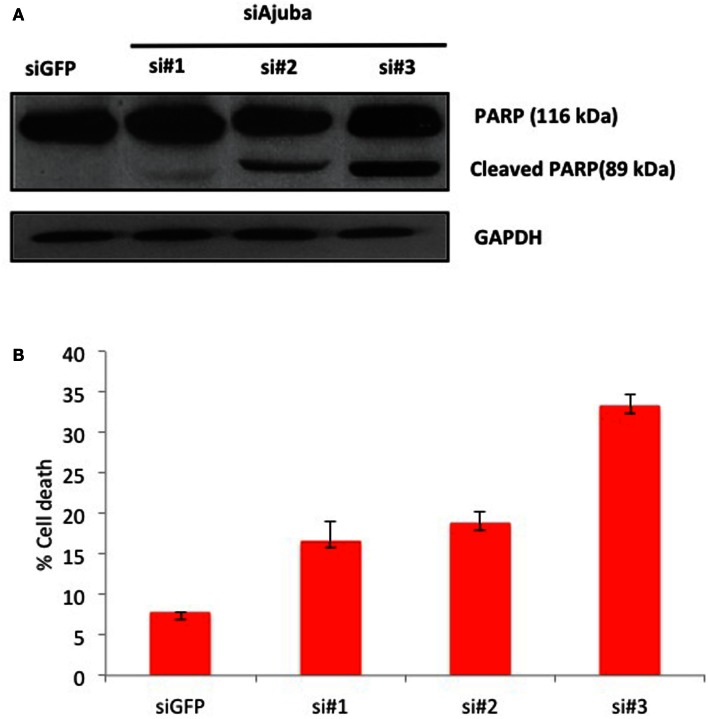
**HTC75 cells depleted for Ajuba undergo apoptosis**. **(A)** Western blot probed with anti-PARP antibody (top), with GAPDH as a loading control (bottom), for the siRNAs indicated on top of the lanes. **(B)** Cell viability measured by Trypan Blue staining, 72 h after transfection with the indicated siRNAs.

We then analyzed the possible activation of apoptosis following Ajuba depletion by probing for PARP, which is cleaved by caspase 3 upon induction of apoptosis. We found extensive accumulation of the PARP cleavage product upon Ajuba depletion, again most significantly with siRNA #3 (Figure 4A). Concomitantly, the number of dead cells doubled for siRNAs #1 and 2, representing 18% of the cell count, compared to 7% with the GFP siRNA control, and topped 30% with siRNA #3 (Figure 4B).

We conclude that the cell death observed was due to the activation of the endogenous apoptotic pathway following ATR and p53 and activation.

### Depletion of Ajuba in IMR90 cells results in a G2/M delay

It was important to analyze the response to Ajuba depletion in another, unrelated cell line in order to establish the importance of the results, and also to address whether the molecular events were specific to tumor cells, or applicable to normal, non-transformed, diploid cells. We chose for this purpose the cell line IMR90, a commonly used primary human fibroblast line. We found that the effects of Ajuba depletion were strikingly similar between HTC75 and IMR90, and not acquired properties as part of a tumor phenotype.

**Figure 5:**
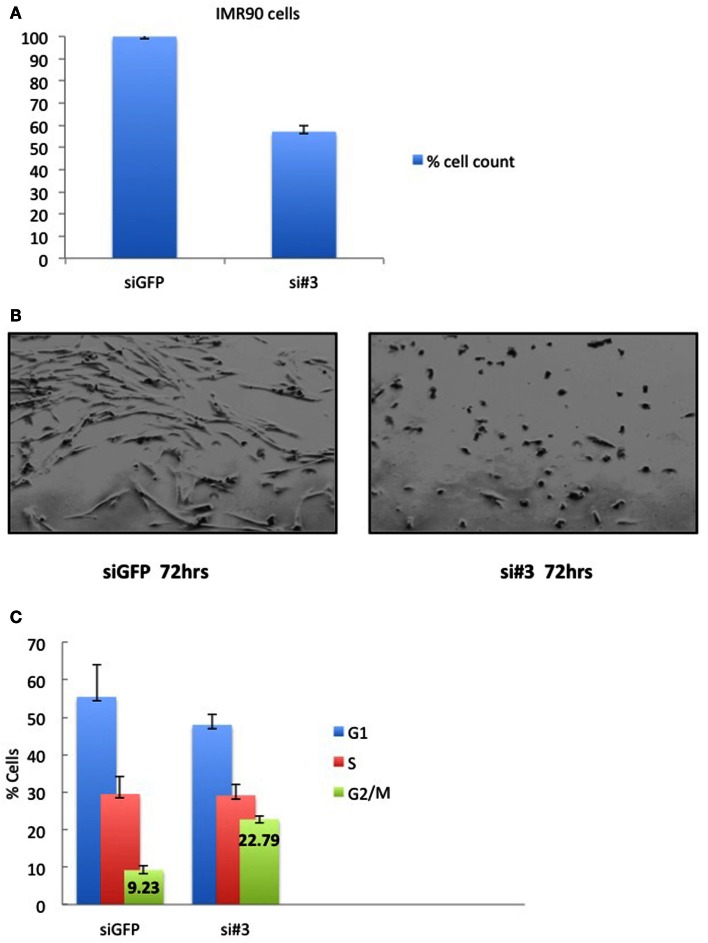
**IMR90 cells depleted for Ajuba have reduced cell number and a G2/M delay**. **(A)** Cell counts of IMR90 cells taken 72 h after transfection of siRNA #3. **(B)** Picture of IMR90 cells for siRNA #3 taken 72 h after transfection. **(C)** Cell cycle profiles of cells processed as in **(B)**, with% of cells in G2/M indicated as averages of three independent experiments.

**Figure 6:**
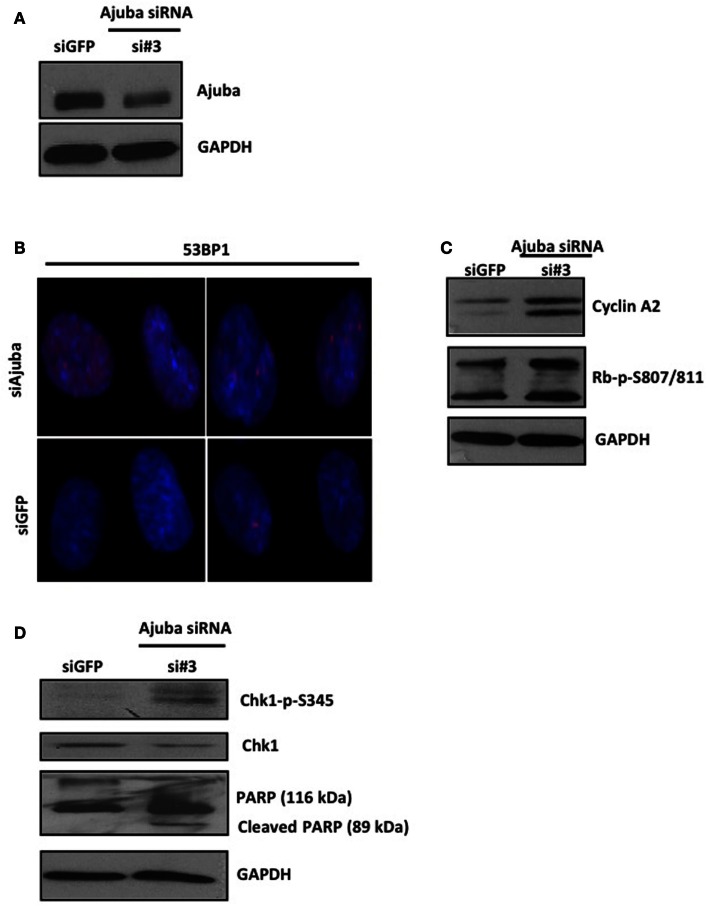
**IMR90 cells depleted for Ajuba undergo a DNA damage response**. **(A,C,D)** Western blots for Ajuba, Cyclin A, Rb, Chk1 phosphorylation, and PARP cleavage on lysates prepared from IMR90 cells 72 h after transfection with siRNA #3. The loading controls with GAPDH for each blot are shown. **(B)** Staining for p53BP1 in Ajuba-depleted cells (siRNA#3), with siGFP as a control.

Depletion of Ajuba in IMR90 cells (Figure 6A) led to a reduction in cell count (Figures 5A,B) and an apparent delay in the cell cycle (Figure 5C). In this case, the delay appeared to be at the G2/M phase (averages of 22.79% against 9.23% in controls) (Figure 5C), corresponding to another known checkpoint for ATR-Chk1 (through Cdc25C, see Discussion). In depleted cells, an increase in Cyclin A2 (Figure 6C), phosphorylation of Chk1 (Figure 6D), and, to a lesser degree, hyperphosphorylation of Rb (Figure 6C), were observed, most prominently with siRNA #3 (not shown). The induction of p53BP1 foci was also evident in Ajuba-depleted cells (Figure 6B). We did detect a low level of PARP cleavage indicating some degree of apoptosis in the cell population (Figure 6D), in accordance with what we observed in HTC75 cells. However, massive apoptosis was not observed nor expected, given the low degree of apoptosis activation in human fibroblasts (Duelli and Lazebnik, 2000). Thus, the nature of the response was highly similar in both cell types analyzed, with, in both cases, an obvious activation of ATR signaling.

We conclude that depletion of Ajuba in IMR90 leads to a qualitatively similar response to our tumor cell system HTC75, which corresponds to ATR activation, but with a different outcome regarding the nature of the cell cycle profile (G2/M delay in IMR90 versus S-phase delay in HTC75). This variation could be due to the different downstream effects of ATR signaling in these two different cell types.

### Ajuba is in a complex with RPA in unperturbed HTC75 or IMR90 cells

**Figure 7:**
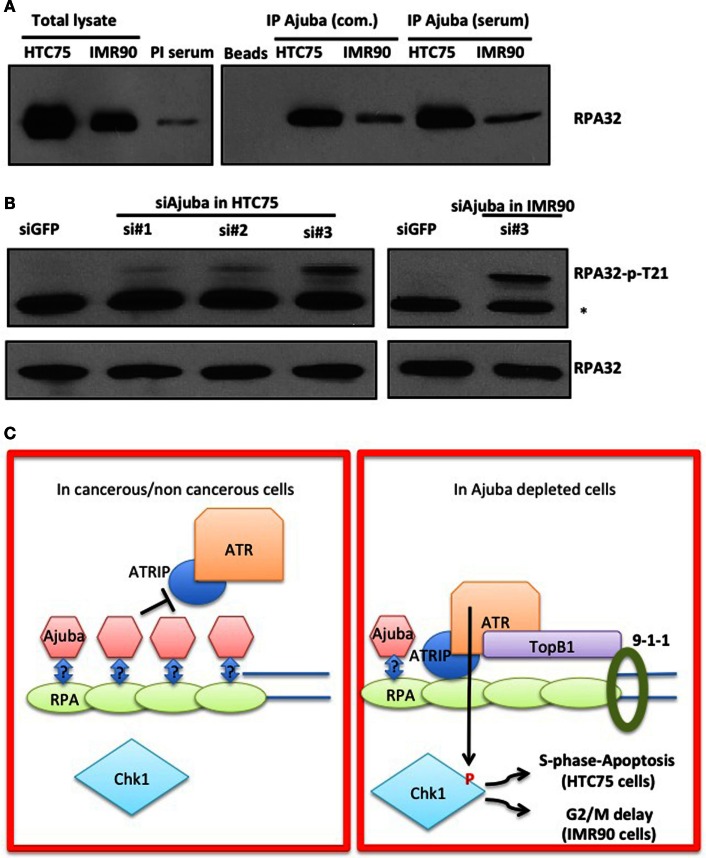
**Ajuba associates with RPA in HTC75 and IMR90 cells**. **(A)** IP-Western probed with a total anti-RPA32 antibody after immunoprecipitation with a commercial anti-Ajuba antibody (com.) or anti-peptide serum from IMR90 or HTC75 extracts as indicated. Left panel: total lysates (input) for each cell line, and the pre-immune serum (PI) used as a control for the immunoprecipitation. **(B)** Western for RPA32-p-Thr21 in both HTC75 and IMR90Ajuba-depleted cells. A non-specific band is marked by a *. Total RPA32 levels are shown at the bottom. **(C)** Model for the role of Ajuba in repression of ATR. The “?” indicates that the interaction between Ajuba and RPA could be direct or indirect. See text for details.

In order to obtain insight on the role of Ajuba in early ATR activation, we reasoned that it could inhibit signaling in the absence of extensive DNA damage during the course of a normal S phase, and that depletion of Ajuba might engage an inappropriately massive response to replication stress. One possibility of how Ajuba exerts its influence on ATR signaling is suggested by our analysis of the role of another, related, LIM protein, TRIP6. We have found that TRIP6 binds POT1 by associating with the OB folds of the protein, and represses the DDR at telomeres (Sheppard and Loayza, 2010). Given the high similarity between TRIP6 and Ajuba, we hypothesized that the Ajuba LIM domains could be dedicated to associate with the RPA OB folds, a known platform for ATR activation (Xu et al., 2008). We tested this hypothesis by asking whether we could immunoprecipitate RPA with Ajuba antibodies (Figure 7A). We found that, in both HTC75 and IMR90 cells, RPA32 could be pulled down with a monoclonal anti-Ajuba antibody as well as with an anti-Ajuba peptide serum, suggesting an interaction between the RPA complex and Ajuba in these cells. Since RPA phosphorylation is required for ATR activation, we hypothesized that Ajuba could prevent this modification. A direct prediction of this model is that, in Ajuba-depleted cells, RPA should be detected as a phosphorylated form indicating activation of the early steps in the ATR pathway. We have tested a monoclonal antibody raised against phosphorylated RPA32-Thr21, known to be PIKK-dependent (Anantha et al., 2007), and found that this form of RPA was significantly increased in Ajuba-depleted cells (Figure 7B). Again, this effect was observed in both cell types used in this study. It would be interesting to test other RPA phosphorylation sites (Liu et al., 2012), such as Ser33 (ATR-dependent) or Ser4/8 (DNA-PK-dependent). We propose a model (Figure 7C), based on our results, in which Ajuba, in unperturbed cells, associates with RPA and protects RPA from unscheduled phosphorylation events, which could lead to an inappropriate ATR response.

## Discussion

This study describes the implication of a novel partner in the DDR, the LIM protein Ajuba. We show here that Ajuba can be described as an inhibitor of the ATR-dependent DDR. This conclusion is based on our observations that depletion of Ajuba leads to a genome-wide DDR which is consistent with ATR activation, such as Chk1 phosphorylation, p53 activation, and induction of p53BP1 foci. The resulting response is a strong overall activation of the pathway as judged by the detection of PARP cleavage, indicating the induction of an apoptotic response. Since these effects are observed in cells that are not experiencing exogenous insults, such as UV irradiation or treatment with drugs inhibiting DNA replication, we argue that Ajuba protects against an unscheduled and excessive response to endogenous DNA damage, which we believe is likely to come from spontaneous replication stress. These possible endogenous DNA damage signals could be the sites of accumulation of p53BP1, possibly representing fork collapses, misincorporated nucleotides, or intra or inter-strand crosslinks for instance. Although our experiments do not address the type of damage eliciting the response, we argue that this damage is normally too weak to activate a full-blown DDR in the presence of Ajuba, but, in Ajuba-depleted cells, can lead to an inappropriate and unscheduled genome-wide response which is lethal to most of the cells and leads to apoptosis. Our results are compatible with other reports (Sørensen et al., 2003; Sorensen et al., 2004) that found the ATR-Chk1-Cdc25A pathway being part of a “surveillance mode” during a normal S phase, but can be activated into an “emergency” DDR after treatment with hydroxyurea, aphidicolin, or UV for instance. We propose here that Ajuba is in this context part of a system that keeps the ATR response in a “surveillance” mode, which could be relieved after extensive exogenous DNA damage. Thus, Ajuba-depleted cells would respond with excessive strength to endogenous and sporadic DNA replication lesions.

We hypothesized that Ajuba plays an important role in inhibiting the DDR mostly in cells of tumor origin due to rampant genome instability and high chromosomal DNA damage in these cells. We therefore analyzed the response in normal human diploid fibroblasts to ask whether the role of Ajuba was important in a context of low level of endogenous DNA damage. Our results show that indeed Ajuba is also important in non-tumor cells to repress the ATR response in absence of exogenous DNA damage. In the diploid fibroblasts we used, the cells responded by a delay in the cell cycle and cell death as well. We observed a qualitative difference in the nature of the cell cycle delay, which corresponded to a G2/M delay in IMR90 cells compared with a S-phase delay in HTC75 cells. The difference in this aspect of the response could be due to the intrinsic wiring of the ATR response in normal fibroblasts, leading to a robust inhibition of Cdc25C and delay of entry into mitosis in these cells. Tumor cells, however, could experience an effect mostly on Cdc25A, which is degraded after Chk1 activation (for review, see Zhou and Bartek, 2004). Degradation of Cdc25A has multiple effects in various parts of the cell cycle, one of them being a strong delay in S phase. More work is required to establish which of Cdc25A or Cdc25C is mostly impacting the cell cycle profiles in either cell types, as well as in other cell types. We think it plausible, although not addressed here, that in both cases the signaling is initiated in S phase in response to DNA replication stress. Thus, the role of Ajuba appears to be important in the context of a normal S phase. In both cell types analyzed here, the response observed is so extensive as to lead to cell death and apoptosis, particularly in the case of the HTC75 cells. Also, this aspect of the response, including activation of p53 and cleavage of PARP, could be under dependence of ATM, known to be activated by a sustained ATR response, and not necessarily a direct effect of the ATR-Chk1 pathway. Such effects have been noted by others (see Hurley and Bunz, 2007).

Overall, these observations imply that ATR is poised for a full-blown response to DNA damage and the pathway requires repression, exerted in part by Ajuba, for progression through the cell cycle, which would allow for local ATR activation at sporadic sites of replication stress such as replication fork collapse or excessive production of single stranded DNA, for instance. Repressors such as Ajuba would keep the response localized and allow for rapid repair and continuation of S phase.

Following the observations linking Ajuba to the repression of the ATR response, we sought to determine the mechanism of action of Ajuba in the ATR activation pathway. We focused on RPA, an essential single strand DNA binding protein constituted by three OB-fold-containing subunits, RPA70, RPA32, and RPA14. RPA has long been documented as playing essential roles in DNA replication, DNA repair and recombination (Wold, 1997), and is an early player in ATR activation following DNA damage (Zou and Elledge, 2003). RPA70 constitutes a platform for the binding of a number of proteins essential for ATR activation, through direct contacts with ATRIP and RAD9, and further recruiting ATR and TOPBP1 (Xu et al., 2008). It is tempting to speculate that Ajuba could bind RPA and prevent ATR activation in undamaged or unstressed cells, thereby preventing the formation of ATR-activating foci. In addition, our laboratory has discovered a similar interaction between POT1, the telomeric overhang binding protein, and the LIM protein TRIP6 (Sheppard and Loayza, 2010). This interaction was initially discovered through a yeast two-hybrid screen, and involved the POT1 N-terminal OB folds and the C-terminal LIM domains of TRIP6. We hypothesized that this domain interaction could be conserved in other protein partners, and in particular between OB-fold-containing RPA and LIM-containing Ajuba. Our results indeed support this hypothesis since RPA could be found in a complex with Ajuba. We are currently following up on this result in asking whether there is a direct interaction between the OB-folds found in RPA and Ajuba in a recombinant protein system. A clear prediction of our model of RPA shielding by Ajuba (Figure 7C) is that, in Ajuba-depleted cells, we should observe increased RPA32 phosphorylation, an early step in ATR activation. Supporting this model, we could clearly detect such an event by looking at RPA32-Thr21 phosphorylation, but more work is required to dissect the exact role of Ajuba in this process. Indeed, the phosphorylation of RPA32 displays a complex pattern, with Thr21 being dependent on ATR itself, Ser33 on ATM and believed to occur during a sustained response, and Ser4 and Ser8 believed to be essential for the early phase of the induction, perhaps even before activation of ATR itself, with DNA-PK as an effector kinase (Liu et al., 2012). Our results show that Ajuba protects from unscheduled Thr21 phosphorylation, definitely placing this molecule at the level of RPA in the repression of the ATR response. Our working model given our results is that Ajuba interacts with the RPA complex and prevents inappropriate phosphorylation of RPA32. It will be interesting to address in the future whether such a role is restricted to S phase or important throughout the cell cycle. Also, whether Ajuba prevents DNA-PK-dependent, or ATR-dependent phosphorylation, or both, remains to be established.

This leaves an important question: how can ATR be activated in the course of DNA replication stress or DNA damage? Possibly, free RPA exists in the cell that could get phosphorylated following such lesions, modifications that could significantly reduce the binding affinity for Ajuba and generate a free, unbound pool able to generate a local ATR response, or a more sustained one depending on the extent of the damage. We are currently testing with recombinant proteins whether the interactions between Ajuba and RPA are direct, and whether specific phosphorylation sites reduce the binding affinities of these interactions, as we would predict. A broader impact of Ajuba and related molecules is that they could have oncogenic properties during early events of cellular transformation by inhibiting the protective or tumor suppressive effects of ATR.

## Conflict of Interest Statement

The authors declare that the research was conducted in the absence of any commercial or financial relationships that could be construed as a potential conflict of interest.

## Abbreviations

ATM, ataxia-telangiectasia mutated; ATR, ataxia-telangiectasia and Rad3-related; Chk1, Checkpoint 1; Chk2, Checkpoint 2; DDR, DNA damage response; FACS, fluorescence activated cell sorting; LIM, Lin-1, Isl-1, Mec-3 domain; RPA, replication protein A.

## References

[B1] Anantha R. W. Vassin V. M. Borowiec J. A. (2007). Sequential and synergistic modification of human RPA stimulates chromosomal DNA repair. J. Biol. Chem. 282, 35910–35923 10.1074/jbc.M704645200 17928296

[B2] Bartkova J. Horejsí Z. Koed K. Krämer A. Tort F. Zieger K. (2005). DNA damage as a candidate anti-cancer barrier in early human tumorigenesis. Nature 434, 864–870 10.1038/nature03482 15829956

[B3] Ciccia A. Elledge S. J. (2010). The DNA damage response: making it safe to play with knives. Mol. Cell 40, 179–204 10.1016/j.molcel.2010.09.019 20965415 PMC2988877

[B4] Duelli D. M. Lazebnik Y. A. (2000). Primary cells suppress oncogene-dependent apoptosis. Nat. Cell Biol. 2, 859–862 10.1038/35041112 11056544

[B5] Gorgoulis V. G. Vassiliou L. V. Karakaidos P. Zacharatos P. Kotsinas A. Liloglou T. (2005). Activation of the DNA damage checkpoint and genomic instability in human precancerous lesions. Nature 434, 907–913 10.1038/nature03485 15829965

[B6] Hurley P. J. Bunz F. (2007). ATM and ATR: components of an integrated circuit. Cell Cycle 6, 414–417 10.4161/cc.6.4.3886 17312392

[B7] Kadrmas J. L. Beckerle M. C. (2004). The LIM domain: from the cytoskeleton to the nucleus. Nat. Rev. Mol. Cell Biol. 5, 920–931 10.1038/nrm1499 15520811

[B8] Karlseder J. Hoke K. Mirzoeva O. K. Bakkenist C. Kastan M. B. Petrini J. H. (2004). The telomeric protein TRF2 binds the ATM kinase and can inhibit the ATM-dependent DNA damage response. PLoS Biol. 2:E240 10.1371/journal.pbio.0020240 15314656 PMC509302

[B9] Lazzerini Denchi E. de Lange T. (2007). Protection of telomeres through independent control of ATM and ATR by TRF2 and POT1. Nature 448, 1068–1071 10.1038/nature06065 17687332

[B10] Liu Q. Guntuku S. Cui X. S. Matsuoka S. Cortez D. Tamai K. (2000). Chk1 is an essential kinase that is regulated by Atr and required for the G(2)/M DNA damage checkpoint. Genes Dev. 14, 1448–1459 10.1101/gad.840500 10859164 PMC316686

[B11] Liu S. Opiyo S. O. Manthey K. Glanzer J. G. Ashley A. K. Amerin C. (2012). Distinct roles for DNA-PK, ATM and ATR in RPA phosphorylation and checkpoint activation in response to replication stress. Nucleic Acids Res. 40, 10780–10794 10.1093/nar/gkr1310 22977173 PMC3510507

[B12] Loayza D. de Lange T. (2003). POT1 as a terminal transducer of TRF1 telomere length control. Nature 424, 1013–1018 10.1038/nature01688 12768206

[B13] Okita N. Minato S. Ohmi E. Tanuma S. Higami Y. (2012). DNA damage-induced CHK1 autophosphorylation at Ser296 is regulated by an intramolecular mechanism. FEBS Lett. 586, 3974–3979 10.1016/j.febslet.2012.09.048 23068608

[B14] Palm W. de Lange T. (2008). How shelterin protects mammalian telomeres. Annu. Rev. Genet. 42, 301–334 10.1146/annurev.genet.41.110306.130350 18680434

[B15] Palm W. Hockemeyer D. Kibe T. de Lange T. (2009). Functional dissection of human and mouse POT1 proteins. Mol. Cell. Biol. 29, 471–482 10.1128/MCB.01352-08 18955498 PMC2612509

[B16] Sheppard S. A. Loayza D. (2010). LIM-domain proteins TRIP6 and LPP associate with shelterin to mediate telomere protection. Aging (Albany N.Y.) 2, 432–444 10.18632/aging.100170 PMC2933890 20634563

[B17] Sheppard S. A. Savinova T. Loayza D. (2011). TRIP6 and LPP, but not Zyxin, are present at a subset of telomeres in human cells. Cell Cycle 10, 1–5 10.4161/cc.10.11.15676 21519191 PMC3142457

[B18] Sørensen C. S. Syljuåsen R. G. Falck J. Schroeder T. Rönnstrand L. Khanna K. K. (2003). Chk1 regulates the S phase checkpoint by coupling the physiological turnover and ionizing radiation-induced accelerated proteolysis of Cdc25A. Cancer Cell 3, 247–258 10.1016/S1535-6108(03)00048-5 12676583

[B19] Sorensen C. S. Syljuasen R. G. Lukas J. Bartek J. (2004). ATR, Claspin and the Rad9-Rad1-Hus1 complex regulate Chk1 and Cdc25A in the absence of DNA damage. Cell Cycle 3, 941–945 10.4161/cc.3.7.972 15190204

[B20] van Steensel B. de Lange T. (1997). Control of telomere length by the human telomeric protein TRF1. Nature 385, 740–743 10.1038/385740a0 9034193

[B21] Vassin V. M. Anantha R. W. Sokolova E. Kanner S. Borowiec J. A. (2009). Human RPA phosphorylation by ATR stimulates DNA synthesis and prevents ssDNA accumulation during DNA-replication stress. J. Cell. Sci. 122, 4070–4080 10.1242/jcs.053702 19843584 PMC2776501

[B22] Wang Y. Gilmore T. D. (2001). LIM domain protein Trip6 has a conserved nuclear export signal, nuclear targeting sequences, and multiple transactivation domains. Biochim. Biophys. Acta 1538, 260–272 10.1016/S0167-4889(01)00077-5 11336797

[B23] Wold M. S. (1997). Replication protein A: a heterotrimeric, single-stranded DNA-binding protein required for eukaryotic DNA metabolism. Annu. Rev. Biochem. 66, 61–92 10.1146/annurev.biochem.66.1.61 9242902

[B24] Xu X. Vaithiyalingam S. Glick G. G. Mordes D. A. Chazin W. J. Cortez D. (2008). The basic cleft of RPA70N binds multiple checkpoint proteins, including RAD9, to regulate ATR signaling. Mol. Cell. Biol. 28, 7345–7353 10.1128/MCB.01079-08 18936170 PMC2593429

[B25] Zhou B. B. Bartek J. (2004). Targeting the checkpoint kinases: chemosensitization versus chemoprotection. Nat. Rev. Cancer 4, 216–225 10.1038/nrc1296 14993903

[B26] Zou L. Elledge S. J. (2003). Sensing DNA damage through ATRIP recognition of RPA-ssDNA complexes. Science 300, 1542–1548 10.1126/science.1083430 12791985

